# Protocol for a randomised controlled trial of a primary care intervention to Reverse Frailty and Enhance Resilience through Exercise and dietary protein Education (REFEREE) in community-dwelling adults aged 65 and over

**DOI:** 10.12688/hrbopenres.13188.2

**Published:** 2021-04-21

**Authors:** John Travers, Roman Romero-Ortuno, Dermot Power, Peter Doran, John Langan, Fergal MacNamara, Darren McCormack, Christopher McDermott, Jude McEntire, Joanne McKiernan, Sebastian Vencken, Andrew W. Murphy, Patrick J. Murphy, Éidin Ní Shé, Diarmuid O'Shea, Marie-Therese Cooney

**Affiliations:** 1 School of Medicine, University College Dublin, Dublin, Ireland; 2 TCD HSE Specialist Training Programme in General Practice, Trinity College Dublin, Dublin, Ireland; 3 Global Brain Health Institute, Trinity College Dublin, Dublin, Ireland; 4 Mercer’s Institute for Successful Aging, St James's Hospital, Dublin, Ireland; 5 Department of Medicine for the Older Person, The Mater Misericordiae University Hospital, Dublin, Ireland; 6 Clinical Research Centre, UCD School of Medicine, University College Dublin, Dublin, Ireland; 7 Health Research Board Primary Care Clinical Trials Network Ireland, National University of Ireland, Galway, Ireland; 8 Discipline of General Practice, National University of Ireland, Galway, Ireland; 9 Department of Geriatric Medicine, St Vincent’s University Hospital, Dublin, Ireland

**Keywords:** Frailty, resilience, sarcopaenia, muscle-mass, primary-care, intervention, exercise, protein, education, randomised controlled trial

## Abstract

**Introduction: **Resistance exercises and dietary protein have been shown to reverse frailty, yet they are not commonly offered in clinical practice. We aim to measure changes in health outcomes, including physical frailty status (SHARE-FI), clinical frailty status (CFS) and muscle mass, as a result of an optimised exercise and dietary intervention versus usual care in a primary care (PC) setting. The intervention has been derived from our systematic review and meta-analysis findings and optimised through patient and public involvement and multidisciplinary team input.

**Methods: **This study is a multicentre randomised controlled parallel arm trial with a three month follow up. 210 eligible people aged 65 and over, no more than mildly frail, will be recruited in seven PC practices in Ireland and randomly assigned to ‘intervention’ or ‘usual care’. Intervention participants will be provided a leaflet with strength exercises, protein dietary guidance and educational discussion. Baseline measurements will include demographics, health indicators, comorbidities, malnutrition universal screening tool (MUST), frailty status (SHARE-FI, CFS) and muscle mass (bioelectrical impedance). Primary outcome will be frailty status measured by SHARE-FI at three months. Secondary outcomes include CFS, muscle mass, in-patient hospitalisation, long term care admission, and subjective ease of intervention and difference to general health. Statistical analysis will be undertaken by an independent statistician.

**Discussion: **The diversity of tested frailty interventions and lack of clear guidance may contribute to low implementation rates. The REFEREE trial focusses on an optimised intervention for a syndrome that poses growing individual and societal challenges. It is hoped results can encourage mainstream adoption of interventions to reverse clinical frailty and build resilience in primary care.

**Trial registration: **ClinicalTrials.gov ID NCT04628754; registered on 13 November 2020.

## Introduction

### Background information

Frailty can be conceptually described as a state of physiological vulnerability to external stressors. It increases the risks of illness, falls, dependency, disability, and death ^1, 2^. Frailty has been described as the most problematic expression of population ageing ^2^.

The challenge of frailty is heightened by our aging population and increased life expectancy. The UN estimates that the population of over 60-year olds will more than double from 2017 to 2050 ^3^. In Ireland, the population of over 65-year-old is growing four times faster than the total population. The prevalence of frailty is estimated at 11% in adults aged ≥50 years and increases to some 50% in those >80 years of age ^4, 5^.

General Practitioners (GPs) are in a unique position to engage with and support their aging patients. Since 2017 the new General Medical Services (GMS) contract in England mandates that all primary care practices use an appropriate tool to identify patients aged ≥65 years who are living with moderate or severe frailty. GPs are also obliged to offer appropriate interventions. Such a mandate to screen and intervene to address frailty is fast becoming international best practice. However, there has been little guidance on the best interventions until recently.

We conducted a systematic review of frailty interventions in primary care, reviewing 925 studies and analysing 46 in detail ^6^. We found that frailty can be delayed and reversed. Results have been further refined and strengthened in our meta-analysis ^7^.

Interventions with both muscle strength training and protein supplementation were consistently placed highest for effectiveness and ease of implementation.

### Rationale for the study

Resistance training done at home and protein supplementation have been shown to be the most effective and easiest to implement interventions to reverse frailty and build resilience. However, it is not common practice to offer and support such interventions in primary care.

Frailty is related to day-to-day symptoms, co-morbidities, disabilities, and overall physical and cognitive functioning. A composite measure of all the latter, guided by the clinician’s clinical judgement, can be assessed by the Clinical Frailty Scale (CFS) ^8^. The use of the CFS has become quite widespread in clinical practice in many countries.

**Figure 1.**
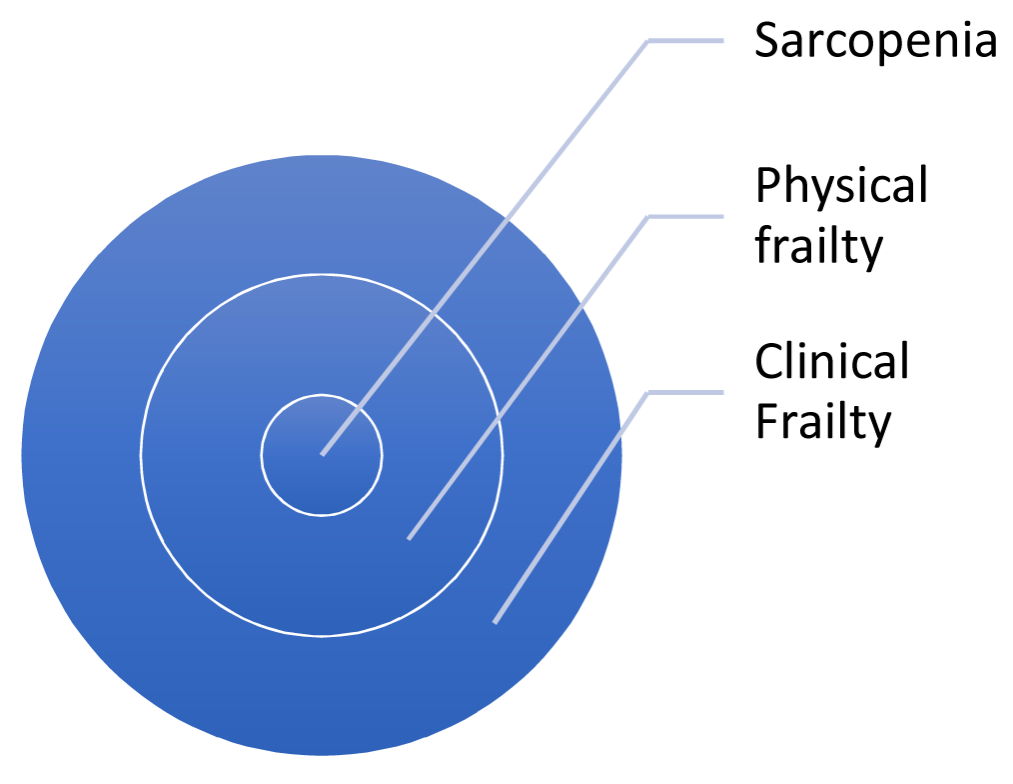
Conceptual framework of the different frailty operationalisations used in this study.

On the other hand, physical frailty status is a narrower concept based on unexplained weight loss, self-reported exhaustion, weakness (by grip strength), slowness (by gait speed) and low levels of self-reported physical activity ^9^. Researchers have adapted the physical frailty definition to allow the provision of a continuous score (e.g. SHARE-FI ^10^). A core component of the physical frailty phenotype is the diagnosis of sarcopenia, which has been operationalised by consensus ^11^ and objectively measured with bioelectrical impedance analysis. Bioelectrical impedance analysis is a safe, non-invasive, rapid and relatively accurate way to assess body composition. Limitations include reduced accuracy for people with BMI > 34 kg/m ^2^, though the technology remains acceptable for monitoring changes in body composition over time. Figure 1 shows the conceptual framework of the different frailty operationalisations used in this study.

Recruitment based CFS seems the most inclusive, but baseline measures will be obtained of all measures at baseline and follow up.

This study provides an opportunity to share an optimised intervention with community-dwelling adults aged 65 and over, whose baseline CFS is not worse than mild (i.e. 5 or less), evaluate improvements in health outcomes and demonstrate how the intervention may be incorporated efficiently in clinical practice. The results are intended to encourage mainstream adoption of practical interventions to reverse clinical frailty and build resilience in primary care.

An intervention with ten recommended resistance exercises and dietary guidance on protein consumption has been derived from findings of our systematic review ^6^ and meta-analysis ^7^ and optimised through a patient and public involvement (PPI) process.

### Feasibility study

Feasibility of the exercise component of the intervention was assessed in a study in 2019 and 2020 ^12^. The findings have been key to refining the intervention for this RCT. We offered an exercise intervention to reverse frailty and build resilience in primary care consultations. We set out to assess uptake of the exercise regime; ratings of its ease and subjective health after participation; how a follow-up telephone-call might affect compliance and overall feasibility. We applied the Bowen feasibility model ^13^ which has been successfully used in assessing public health and preventive medicine interventions. This feasibility model tests eight areas of focus: acceptability; demand; implementation; practicality; adaptation; integration; expansion; and limited-efficacy testing. Selected results include:

A total of 94 of 107 eligible people (88%) participated (average age 77, 59 females (63%)). 95% of females and 78% of males enrolled. Only 15% had previously considered resistance exercises.

Participants found the exercises both easy to follow and generally easy to do. Overall, 32% described exercises as ‘very easy’, 55% ‘somewhat easy’, 7% ‘neither easy nor hard’, 6% ‘somewhat hard and 0% ‘very hard’.

At one month, 66% of participants called were exercising regularly (76% of females, 50% of males). At two months, compliance among those who had not been called at one month was: 65% (67% of females, 62% of males). However, compliance among those previously called was higher: 78% (88% of females, 63% of males). 64% of those who were not doing exercises at one month had taken them up following the single phone call (67% of females, 63% of males).

Many described how the home-based exercises helped with staying active and reducing anxiety while housebound during the Covid-19 pandemic

The majority of participants self-reported their general health had improved as a result of doing the exercises and none reported feeling worse. 14% felt ‘much better’, 52% ‘slightly better’, 34% ‘about the same’, 0% ‘slightly worse’, and 0% ‘much worse’

Each of the eight areas of focus of the Bowen feasibility model have been addressed and satisfy overall feasibility. Publication of this feasibility study is pending.

### Public and patient involvement

Public and patient involvement in health involves undertaking research with members of the public rather than for them. As PPI has been a key consideration within Irish health research culture, we wanted to enable the involvement of potential participants’ in co-designing our RCT to ensure that their priorities were at its core. We built on two group discussions with 24 over 65-year olds, when introducing an exercise regime in a previous service evaluation study ^14^, and 145 one-on-one discussions listening to feedback during the feasibility study. We wished to learn additionally from participants in three online workshops to optimise our patient communication model, refine the exercise regime and develop dietary guidance for a future trial.

We convened three online group workshops with older adults involved in the feasibility study. The group size of an average of 5 participants and 2 researchers was in keeping with best practice for conducting both group-based action learning ^15^ and focus group activity ^15, 16^. Key themes and questions for each workshop were prepared in advance. A Socratic approach of open questions with active listening was used in order to ensure the voices of the non-researchers are primarily heard. Transcripts were analysed and key feedback synthesised under the headings of: ‘patient engagement and communication model’, ‘exercise regime’ and ‘dietary guidance’. Analysis used both an inductive and deductive approach drawing on normalisation process theory. Our approach was guided by a framework for effective and meaningful public and patient representative (PPR) involvement in health research ^17– 19^, which includes operating in a structured environment; managing expectations and ensuring roles are clear; providing support for successful participation and ensuring inclusive representation; and commitment to co-learning involving institutional leadership.

PPI output included strong endorsement from participants for offering this intervention; co-design of the patient communication model; inclusion of preferred setting, medium and gender balance for the exercise regime; and co-design of the dietary guidance content.

### Benefits and risks

The systematic review ^6^ identified benefits to patients that include decreased risks of illnesses, falls, dependency, disability and improved mortality. 71% of studies measuring impact on frailty status demonstrated significant improvement. 69% of studies measuring impact on singular frailty indicators or other criteria demonstrated significant improvement. The exercises create a small risk of musculoskeletal strain. No such adverse event was recorded during a feasibility study of the intervention, prior to PPI optimisation. The risk was mitigated by the fact that the exercises are simple in nature and the supporting patient information leaflet advises thirty second breaks between exercises and not to undertake any exercise that causes pain. Risk is further mitigated by excluding participants with CFS 6 and above. The risk (probability) of harm is considered to be low.

## Study objective

### Primary objective

To measure changes in health outcomes, including physical frailty status (SHARE-FI), clinical frailty status (CFS), muscle-mass, as a result of an optimised exercise and dietary intervention versus usual care among community-dwelling adults aged 65 and over whose baseline clinical frailty (CFS) is not worse than mild.

### Secondary objective

To understand participants’ perspectives on the ease of the intervention and personal health benefits.

### Primary and secondary endpoints/outcome measures

***Primary outcomes***

- Frailty status measured by SHARE-FI at three months after the start of intervention.

***Secondary outcomes***

- Clinical frailty status measured by CFS at three months after the start of intervention.
- Muscle mass measured by bioelectrical impedance at three months after the start of intervention.
- Body fat, bone weight, base metabolic rate, biological age by bioelectrical impedance at three months after the start of intervention.
- Incidence of in-patient hospitalisation during follow-up.
- Incidence of long-term care admission (LTC) during follow-up.
- Ease of the intervention measured on a five-point scale: ‘very easy’, ‘somewhat easy’, ‘neither easy nor hard’, ‘somewhat hard’, or ‘very hard’ three months after the start of intervention.
- Difference to general health as a result of the exercises measured on a five-point scale: ‘much better’, ‘slightly better’, ‘about the same’, ‘slightly worse’ or ‘much worse’ three months after the start of intervention.

## Trial design

### General considerations

**Figure 2.**
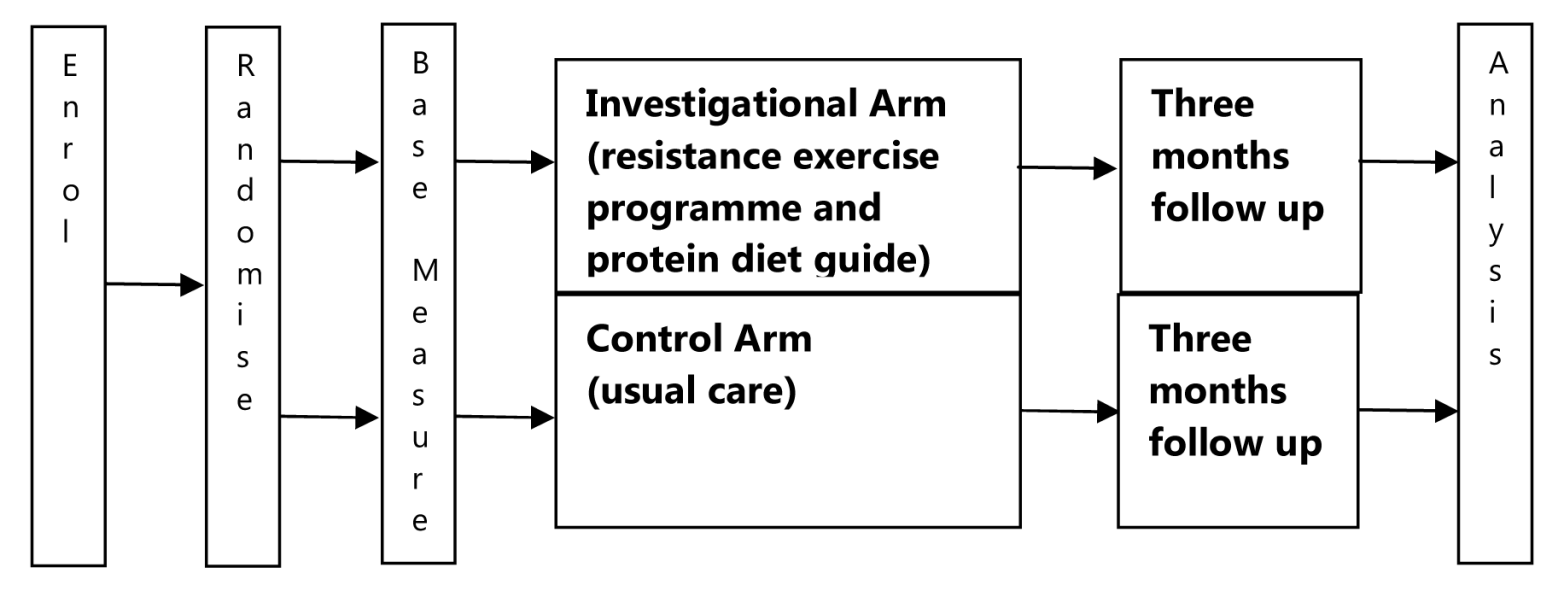
Study schema.

This multi-centre, randomised controlled, parallel arm trial aims to measure the effectiveness of an optimised primary care intervention to reverse frailty and build resilience versus usual care, among pre-frail and mildly frail adults aged 65 and over. The trial will take place in Ireland. Enrolment will take place in seven GP practices in Dublin and Wicklow over a period of six months. All measures will be collected in the GP practice setting. A clear training plan and documentation will be provided to GPs at each site to mitigate the risk of recorded and unrecorded protocol deviations ^20^. A study scheme is shown in Figure 2.

### Selection of study population

All patients aged 65 or older attending the GP site investigator will be screened for eligibility by the GP. Patients meeting all of the inclusion criteria and none of the exclusion criteria will be eligible for inclusion.

***Inclusion criteria***

- Aged 65 or older at baseline
- Rockwood clinical frailty scale score 4 or 5 (vulnerable or mildly frail)
- Able and willing to provide informed consent and to comply with the requirements of this study protocol

***Exclusion criteria***

- Rockwood clinical frailty scale score > 5
- End of life care
- Persons in residential care home
- Concurrent malignancy
- Chronic kidney disease, stage 3 or 4
- Coded diagnosis of severe dementia as per GP or consultant geriatrician diagnosis or baseline Montreal Cognitive assessment (MoCA) score ≤ 10
- Persons unable to engage in discussion on frailty due to acute care needs or determined to be inappropriate by GP (e.g., needing transfer to ED or acutely unwell or disorders resulting in intolerance of the intervention)
- Subjects unable to provide written informed consent

### Study methods, assessments and procedures

GP site investigators will undergo three team-based training sessions prior to enrolment, monthly meetings after enrolment and additional ad-hoc meetings when required, led by the principal investigator, in order to standardise the approach and align to this study protocol, including patient communication, intervention delivery and data gathering and management.

Adults attending a GP surgery, meeting the eligibility criteria, will be offered general information about the study by the attending GP. If interested and willing to consent, they will be offered an information leaflet for the trial, and invited to participate. Participants will be randomly assigned to ‘intervention’ or ‘usual care’ parallel arms.

Participants randomly assigned to the usual care group will receive normal primary care, including physical rehabilitation if needed.

Intervention participants will be provided a leaflet with strength exercises and a discussion on how strength exercises have been shown to delay and reverse frailty and build resilience. The information leaflet will have a photographic overview of a home-based exercise regime. They will also be provided with information on post exercise protein consumption as part of a normal balanced diet.

Baseline measurements for all recruited patients will be: age, sex, education, living arrangement (alone, with others), smoking status, alcohol intake, blood pressure, heart rate, BMI (weight and height), comorbidities, malnutrition universal screening tool (MUST), CFS, SHARE-FI (which includes handgrip strength) and bioelectrical impedance for muscle mass, body fat, bone weight, base metabolic rate and ‘biological age’. Bioelectrical impedance will be measured using a Tanita RD 545 Segmental Body Composition Analyser, with dual frequency technology and medical grade accuracy.

**Figure 3.**
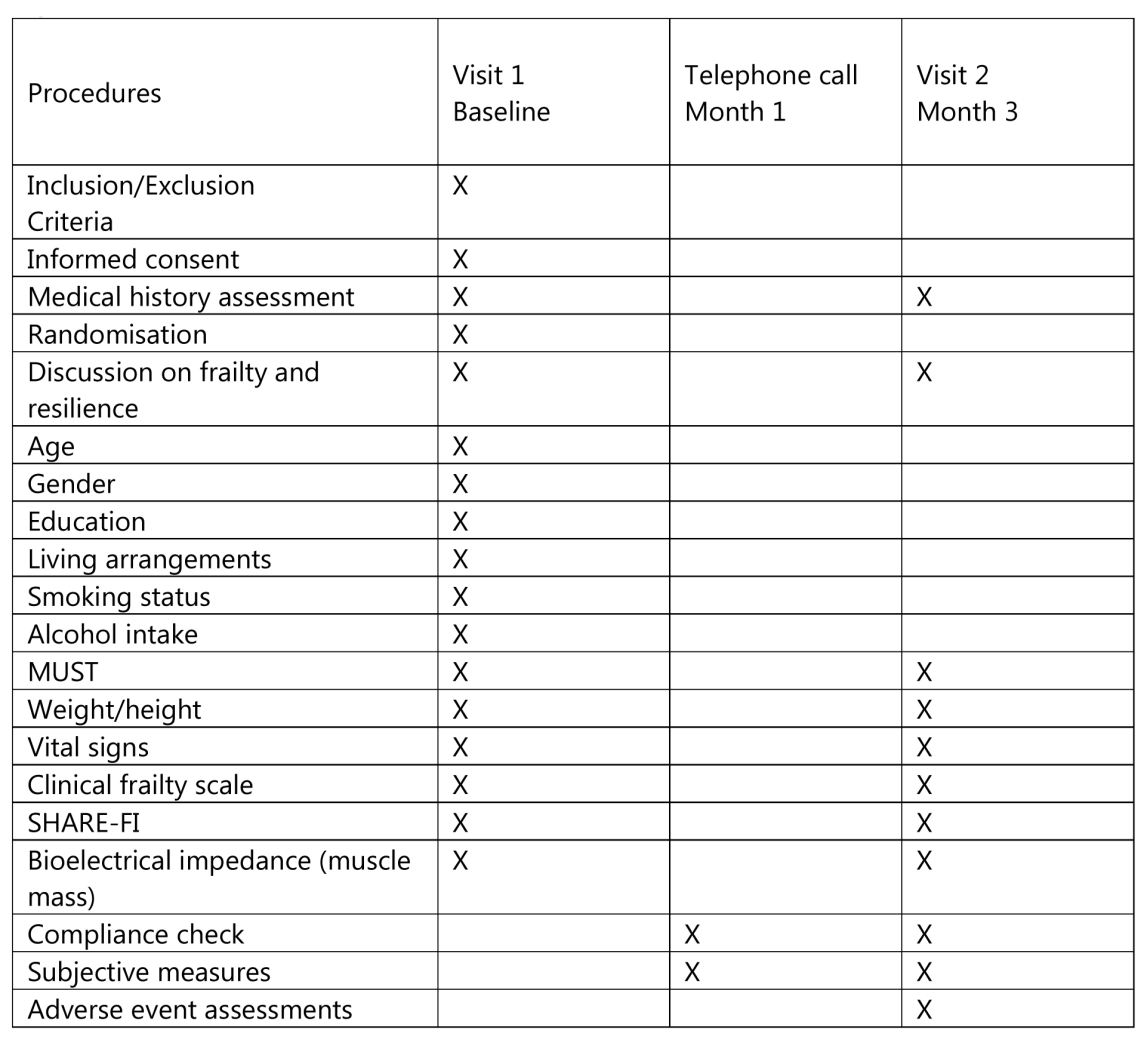
Schedule of events.

Intervention participants will be called after one month and after three months by the GP and asked if they had followed the exercise regime (and for how long and how many times a week) and dietary guidance. At three months they will be asked if they found the exercises ‘very easy’, ‘somewhat easy’, ‘neither easy nor hard’, ‘somewhat hard’, or ‘very hard’; and had they noticed any subjective difference to their health as a result of the intervention, namely feeling ‘much better’, ‘slightly better’, ‘about the same’, ‘slightly worse’ or ‘much worse’. All participants will be invited to return at three months for a clinical assessment and measurement of health indicators and frailty status as described above by the GP. Participants will be invited to return for subsequent one year follow up appointments. This 12-month follow up will be subject to a further ethics committee review. The follow up visits will be free of charge. A schedule of events for baseline, one-month call and three months follow up are shown in Figure 3.

### Data management

Data will be recorded in a pseudo-anonymised form in a password protected database. Documents containing personal information and signed consent forms will be kept in a locked file at the principal investigator’s office at each centre. Data will be added to the electronic database by authorised team members only and access will be limited to authorised team members. No participant will be identified or will be identifiable as a result of data processing or in any subsequent publication. Data will be destroyed 5 years after the end of the study or 3 years after the last publication as per the guidelines described by the research ethics committee of the Irish College of General Practitioners. The lead PI (JT) will be the data controller. Data processors will be the research team members, including the 6 co-investigators in each of the centres.

### Method of assigning participants to treatment groups

***Randomisation*.** Randomisation of participants will be done on a 1:1 allocation to intervention or usual care by a simple randomisation procedure, guided by the Clinical Research Centre (CRC) team. We will use a randomisation software product provided by the American National Cancer Institute.

### Definition of end-of-trial

The end of trial will be the date of the last visit of the last subject. The trial team has the right at any time to terminate the study for clinical or administrative reasons. The end of the study will be reported to the REC within 90 days. A summary report of the study will be provided to the REC and Regulatory Authority within 1 year of the end of the study.

***Premature termination of the study*.** The trial team may end the study pre-maturely on the basis of new information about safety of the intervention or unsatisfactory progress in enrolling participants (i.e., <50% of sample size after 6 months).

### Discontinuation/withdrawal of subjects from study protocol

Subjects have the right to voluntarily discontinue the intervention or withdraw from the study at any time for any reason without any consequences. The investigator has the right to discontinue a subject from study treatment or withdraw a subject from the study at any time if it is in the best interest of the subject.

Subjects must discontinue the intervention and be withdrawn from the study for any of the following reasons:

-   withdrawal of consent by the subject

-   any medical condition that the investigator or sponsor determines may jeopardize the subject’s safety if she or he continues receiving the study treatment

-   ineligibility (either arising during the study or retrospectively having been overlooked at screening)

-   an adverse event which requires discontinuation of the intervention

-   lack of compliance with the study and/or study procedures

-   lost to follow-up. At least three documented attempts must be made to contact any subject lost to follow-up.

If a subject is withdrawn before completing the study, the reason for withdrawal must be documented. If a subject is withdrawn due to an adverse event, the investigator will arrange for follow-up visits until the adverse event has resolved or stabilised.

## Intervention

### Description of intervention

**Figure 4.**
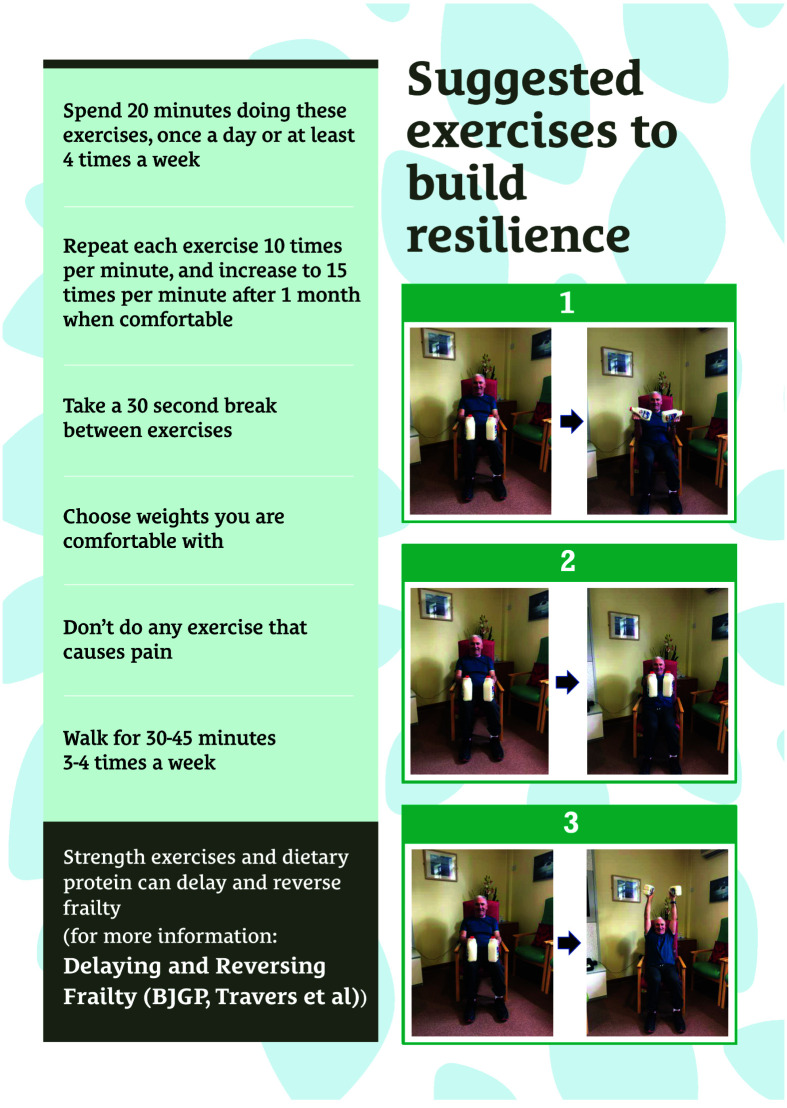
Sample exercise leaflet.

**Figure 5.**
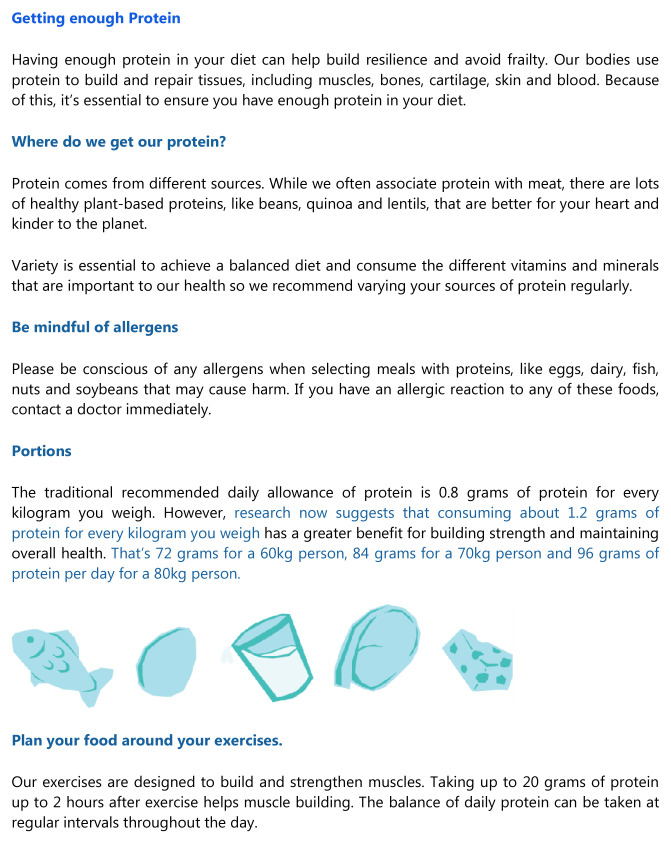
Sample dietary protein guidance leaflet.

The resistance exercise regime consists of ten, home-based, physical exercises as illustrated in an accompanying leaflet. Each exercise is to be repeated ten times in one minute, increased to fifteen times per minute after about 1 month, when comfortable. A break of 30 seconds is to be taken between sets of exercises. Exercises are to be undertaken at least four times per week and up to every day. Participants should also walk for 30 to 45 minutes, three to four times per week. Participants are advised to consume 1.2 g protein per kg body weight each day. A leaflet will be provided with information on sources of protein, amounts of protein per typical serving and suggested meals. A sample exercise leaflet and protein dietary guidance are shown in Figure 4 and Figure 5.

## Safety reporting

Participants are asked not to undertake any exercise that causes pain and to discontinue the exercise regime if they have any concern, injury or physical signs constraining participation. Safety will be evaluated throughout the study by recording adverse events (AEs), and inviting participants to attend for assessment of vital signs and physical exam, should they contact their GP with any concerns.

### Definitions

***AE*.** Any untoward medical occurrence in a participant and which does not necessarily have a causal relationship with this intervention. An adverse event can therefore be any unfavourable and unintended sign, symptom or disease, whether or not considered related to the intervention.

***Serious AE (SAE)***

Any untoward medical occurrence or affect that:

-   results in death,

-   is life-threatening*,

-   requires hospitalisation or prolongation of existing hospitalisation,

-   results in persistent or significant disability or incapacity,

-   is a congenital anomaly or birth defect

-   important medical events**

*Regarding a life-threatening event, this refers to an event in which the subject was at risk of death at the time of the event; it does not refer to an event which hypothetically might have caused death if it were more severe.

**Some medical events may jeopardise the subject or may require an intervention to prevent one of the above characteristics/consequences. Such events (hereinafter referred to as ‘important medical events’) should also be considered as ‘serious’ in accordance with the definition

***Severity of AEs*.** The term ‘severity’ is used here to describe the intensity of a specific event. This has to be distinguished from the term ‘serious.

### Evaluation of AEs and SAEs

***Assessment of seriousness*.** The investigator should make an assessment of seriousness.

***Assessment of casualty*.** The investigator/sponsor must make an assessment of whether the AE/SAE is likely to be related to the intervention according to the following definitions:

Unrelated

Where an event is not considered to be related to the study intervention.

Possibly

Although a relationship to the study intervention cannot be completely ruled out, the nature of the event, the underlying disease, or temporal relationship make other explanations possible.

Probably

The temporal relationship and absence of a more likely explanation suggest the event could be related to the study intervention.

All AEs/SAEs judged as having a reasonable suspected causal relationship (e.g. possibly, probably) to the study intervention will be considered as ARs/SARs.

All AEs/SAEs judged as being related (e.g. possibly, probably) to an interaction between the study medication and another medication will also be considered to be ARs/SAR.

Alternative causes such as natural history of the underlying disease, concomitant therapy, other risk factors and the temporal relationship of the event to the treatment should be considered.

***Assessment of severity*.** The investigator will make an assessment of severity for each AE/SAE and record this on the CRF according to one of the following categories:

Mild

An event that is easily tolerated by the subject, causing minimal discomfort and not interfering with everyday activities.

Moderate

An event that is sufficiently discomforting to interfere with normal everyday activities.

Severe

An event that prevents normal everyday activities.

### Reporting procedures for all adverse events

All AEs occurring during the study observed by the investigator or reported by the subject, whether or not attributed to the study medication, will be documented.

The following information will be recorded: description, date of onset and end date, severity, assessment of relatedness to the study intervention, other suspect medication or device and action taken. Follow-up information should be provided as necessary.

AEs considered related to the study intervention as judged by an investigator will be followed until resolution or until the event is considered stable. All related AEs that result in a subject’s withdrawal from the study or are present at the end of the study, should be followed up until a satisfactory resolution occurs.

It will be left to the investigator’s clinical judgment whether or not an AE is of sufficient severity to require the subject’s removal from the study. A subject may also voluntarily withdraw due to what he or she perceives as an intolerable AE. If either of these occurs, the subject must undergo an end-of-study assessment and be given appropriate care under medical supervision until symptoms cease or the condition becomes stable.

The severity of events will be assessed on the following scale: mild, moderate, severe.

The relationship of AEs to the study intervention will be assessed by the investigator.

### Reporting procedures for serious adverse events

The investigator will report all serious adverse events immediately to the supervisor. The immediate report will be followed by detailed, written reports. The immediate and follow-up reports will identify subjects by unique code numbers assigned to the latter.

The immediate report will be made by the investigator within a very short period of time and under no circumstances should this exceed 24 hours following knowledge of the serious adverse event.

The supervisor will keep detailed records of all adverse events which are reported to her by the investigator or investigators.

## Statistics

### Description of statistical methods

Final analysis of the trial will be performed by the independent trial statistician after end-of-trial.

### Determination of sample size subjects

We have calculated a sample size of 176 with the following parameters: Two independent study groups (i.e., control and intervention); outcome measure of frailty status (i.e., change from frail or pre-frail to non-frail as measured by SHARE-FI); improvement of frailty status in the intervention group to non-frail of 15%, allowing for a 3% improvement in the control group due to bias; enrolment ratio of 1; probability of type I error of 5% (0.05); power 80%. Enrolment of 210 participants assumes a 15% drop out rate.

Sample sizes in comparable trials include Binder, J Am Geriat Soc 2002 (sample size 115) ^20^, Liu, Clin Rehab 2017 (sample size 79) ^21^ and Serra-Prat, Age Aging 2017 (sample size 172) ^2^. The average study size of the 31 studies included in our meta-analysis of frailty interventions ^7^ was exactly 100.

### Analysis sets

Final analysis for this trial will be conducted on the intention-to-treat population, which will include all subjects randomised to the treatment arms. Sensitivity analysis will be conducted on the per protocol population (full analysis set), which will include all subjects that attend all study visits and have complied with the intervention.

### Demographic and baseline disease characteristics

Baseline descriptive statistics of the study cohort will be stratified by arm, with the sample sizes reported for each arm. Mean (standard deviation) and median (interquartile range) will be reported for numerical outcomes as appropriate. Frequency and proportion will be reported for categorical outcomes. These statistics will be reported for both the intention-to-treat population and the per protocol population.

### Efficacy analysis

Efficacy analysis will be performed on all primary and secondary efficacy endpoints. Point estimates, interval estimates (95% confidence interval) and p-values will be reported.

***Primary efficacy endpoint*.** The primary outcome is Frailty status measured by SHARE-FI at three months of follow-up.

Changes in proportions in non-frail, pre-frail and frail categories will be analysed in both study arms and reported as an odds ratio with associated 95% confidence intervals and tested for significance using a standard chi-square test of proportions.

We hypothesise that participants on resistance exercise regime and with guidance on dietary protein will have a higher SHARE-FI score than patients without this intervention.

This outcome will be analysed as a difference in means between treatment arms using a linear mixed effects model, with arm allocation, SHARE-FI at baseline and age included as fixed effects and GP practice included as a random effect.

***Secondary efficacy endpoints*.** Clinical frailty status measured by CFS at three months will be analysed using a proportional odds mixed model with arm allocation and age as fixed effects and GP practice as random effect. Muscle mass measured by bioelectrical impedance at three months will be analysed using a logistic mixed model with arm allocation as fixed effect and GP practice as random effect.

Ease of the intervention measured on a five-point scale and difference to general health as a result of the exercises measured on a five-point scale will be analysed using a proportional odds model with arm allocation as covariate.

### Safety analysis

Incidence of in-patient hospitalisation during follow-up and incidence of LTC during follow up will be analysed using a logistic regression model with arm allocation and age as covariates.

All non-serious and serious AEs will be reported in tables in aggregate form by frequency and subjects affected by trial arm.

### The level of statistical significance

For hypothesis testing the significance level will be set at 0.05.

### Procedure for accounting for missing, unused and spurious data

Where appropriate, missing data will be imputed using multivariate imputation by chained equations (van Buuren & Groothuis-Oudshoorn, 2011).

### Procedure for reporting any deviation(s) from the original statistical plan

Deviation(s) from the original statistical plan will be reported in writing to the research ethics committee and approval sought.

## Retention of essential documents

All records and documents will be maintained by the principal investigator for a period of 12 months after the end of the trial.

## Ethics

### Declaration of Helsinki

The supervisor will ensure that this study is conducted in accordance with the ethical principles that have their origins in the Declaration of Helsinki.

### Good clinical practice

This study will be conducted in accordance with Good Clinical Practice, as defined by the International Conference on Harmonisation (ICH) and in accordance with the ethical principles underlying European Union Directive 2001/20/EC and 2005/28/EC.

### Approvals

Ethical approval has been granted by the research ethics committee of the Irish College of General Practitioners on November 3, 2020

Approval for protocol modifications or amendments will be sought through formal REC submission and communicated to stakeholders (e.g., investigators, REC/IRBs, trial participants, trial registries, journals) by email where appropriate or by phone to participants with consent.

### Informed consent

The GP will take informed consent in the primary care consultation. Informed consent will be obtained prior to any study related procedures being undertaken. The GP will explain the nature of the study to the participant, and answer all questions regarding this study. Prior to any study-related screening procedures being performed, the informed consent statement will be reviewed and signed and dated by the participant and the GP.

A model consent form is available at the Harvard Dataverse repository ^22^.

### Subject confidentiality

The trial staff will ensure that the participants’ anonymity is maintained. The participants will be identified only by first names and identification number on any database. All documents will be stored securely. The study will comply with the Data Protection Act.

### Data management committee

In line with guidance from the European Medicines Agency, an external data management committee will not be part of this trial, due to the short time frame, non-critical nature of the syndrome being addressed and low risk of harm from the intervention.

## Insurance/Indemnity

The GPs hold medical liability insurance as required by the Irish College of General Practitioners and the Medical Council.

## Dissemination

Initial dissemination of results will be through publication in a peer reviewed journal. The study team will also work with advocacy, primary care and academic partners to disseminate the findings. These will include, Age Action, the Irish College of General practitioners, University College Dublin and Trinity College Dublin. The study is at an early stage and is due to commence enrolment in December 2020. However, early planning for dissemination is underway and agreement for same received from the Health Research Board Primary Care Clinical Trials Network.

## Study management

The principal investigator is John Travers. The lead investigators at GP practices are John Langan, Darren McCormack, Jude McEntire, Fergal MacNamara, Chris McDermott, Joanne McKiernan and John Travers. Marie-Therese Cooney, consultant geriatrician at St Vincent’s University Hospital and associate professor at UCD will supervise the study. Peter Doran, professor of medicine and associate dean of research at UCD, and scientific director of the UCD clinical research centre (CRC), will co-supervise the project. Dermot Power, consultant geriatrician at the Mater Misericordiae University Hospital and professor of medicine at UCD, will co-supervise the project. Roman Romero-Ortuno, consultant geriatrician and associate professor at the TCD Global Brain Health Institute, will help with study design, implementation and writing.

## Data availability

### Underlying data

All data underlying the results are available as part of the article and no additional source data are required.

### Extended data

Harvard Dataverse: Reversing Frailty and Enhancing Resilience (REFEREE) RCT. https://doi.org/10.7910/DVN/RKEGIV ^22^.

This project contains a model consent form used in this study.

### Reporting guidelines

Harvard Dataverse: SPIRIT checklist for ‘Protocol for a randomised controlled trial of a primary care intervention to Reverse Frailty and Enhance Resilience through Exercise and dietary protein Education (REFEREE) in community-dwelling adults aged 65 and over’. https://doi.org/10.7910/DVN/TCOCSV ^23^.

Data are available under the terms of the Creative Commons Attribution 4.0 International license (CC-BY 4.0).
