# Supplementary material for: Protocol for a randomised controlled trial of a primary care intervention to Reverse Frailty and Enhance Resilience through Exercise and dietary protein Education (REFEREE) in community-dwelling adults aged 65 and over
Source: HRB Open Res. 2021 Apr 21;3:91. Originally published 2020 Dec 21. [Version 2] doi: 10.12688/hrbopenres.13188.2 (PMC8080984; doi:10.12688/hrbopenres.13188.2)
Supplement: Peer review history

# Peer review: Reviewer response for version 2

**DOI:** 10.21956/hrbopenres.14426.r29307

**Published:** 2021-04-22

**Authors:** Nicolas Martinez-Velilla

**Affiliations:** 1 Navarrabiomed, Complejo Hospitalario de Navarra (CHN), Navarra Health Research Institute (IdisNa), Public University of Navarra (UPNA), Navarra, Spain

Dear authors, congratulations. You have addressed all my concerns.

Is the study design appropriate for the research question?

Yes

Is the rationale for, and objectives of, the study clearly described?

Yes

Are sufficient details of the methods provided to allow replication by others?

Yes

Are the datasets clearly presented in a useable and accessible format?

Not applicable

Reviewer Expertise:

NA

I confirm that I have read this submission and believe that I have an appropriate level of expertise to confirm that it is of an acceptable scientific standard.

# Response

**Published:** 2021-04-22

**Authors:** John Travers

**Affiliations:** University College Dublin, Ireland

Dear Dr Martinez-Velilla, Thanks so much for your great support and feedback. I wish you every continued success with your work. Best wishes, John

# Peer review: Reviewer response for version 2

**DOI:** 10.21956/hrbopenres.14426.r29306

**Published:** 2021-04-21

**Authors:** Rose Galvin

**Affiliations:** 1 School of Allied Health, Ageing Research Centre, Health Research Institute, University of Limerick, Limerick, Ireland

I welcome the authors responses to my comments and their endeavours at integrating them into the manuscript. I have no further comments to make.

Is the study design appropriate for the research question?

Yes

Is the rationale for, and objectives of, the study clearly described?

Yes

Are sufficient details of the methods provided to allow replication by others?

Yes

Are the datasets clearly presented in a useable and accessible format?

Yes

Reviewer Expertise:

Ageing, frailty, research methods

I confirm that I have read this submission and believe that I have an appropriate level of expertise to confirm that it is of an acceptable scientific standard.

# Response

**Published:** 2021-04-21

**Authors:** John Travers

**Affiliations:** University College Dublin, Ireland

Thank you Dr Galvin for all your guidance and feedback. Wishing you every success with your work. John

# Peer review: Reviewer response for version 1

**DOI:** 10.21956/hrbopenres.14324.r28582

**Published:** 2021-02-22

**Authors:** Rose Galvin

**Affiliations:** 1 School of Allied Health, Ageing Research Centre, Health Research Institute, University of Limerick, Limerick, Ireland

I am delighted with the opportunity to review the protocol for a randomised controlled trial of a primary care intervention to Reverse Frailty and Enhance Resilience through Exercise and dietary protein Education (REFEREE) in community-dwelling adults aged 65 and over. It is welcome to see interventions for frailty delivered in the community.  

 The rationale for conducting the current study is well articulated and the manuscript adheres to the relevant standardised reporting guidelines. There are a few issues in the methods that warrant further clarification.

 In the methods section, it would be helpful if the authors justified the exclusion criteria (cut-off on CFS). Furthermore, the process around screening and recruitment could be clearer. Will the practice nurse play a role in screening/recruitment? Who will administer the CFS at baseline?

 Who will complete the follow-up assessments across at the sites and how will it be standardised across sites?

 The authors should justify their rationale to destroy the data 5 years after the end of study.

 The plan to disseminate the study findings is very comprehensive.

 Overall, the REFEREE trial protocol describes an intervention for frailty that is evidence-based and stakeholder informed. The pragmatic intervention focuses on a syndrome that poses growing individual and societal challenges.

Is the study design appropriate for the research question?

Yes

Is the rationale for, and objectives of, the study clearly described?

Yes

Are sufficient details of the methods provided to allow replication by others?

Yes

Are the datasets clearly presented in a useable and accessible format?

Yes

Reviewer Expertise:

Ageing, frailty, research methods

I confirm that I have read this submission and believe that I have an appropriate level of expertise to confirm that it is of an acceptable scientific standard, however I have significant reservations, as outlined above.

# Response

**Published:** 2021-03-10

**Authors:** John Travers

**Affiliations:** University College Dublin, Ireland

Thank you very much for this very helpful and constructive feedback, which has enabled us to improve our approach to this trial. We have addressed each of these points in the text below and a revised protocol. We look forward to any other feedback that you may have.

 1) People who are estimated to be at CFS level 6 and above require varying levels of assistance. The exercise intervention, derived from our systematic review and meta-analysis and co-designed by PPI, is for daily, independent use in the home, without assistance or carer supervision. We wish to minimise risk to participants and therefore feel it is appropriate to exclude a level of frailty that corresponds to CFS 6 and above. We have added this to the study rational (page 5). We plan to consider appropriate interventions for people with greater degrees of frailty in our future work.

 2) Thank you, we have amended the protocol to make the process around screening and recruitment clearer (page 8). The GP co-investigator will undertake screening and recruitment at each site. In order to avoid inter-operator variability, the GP will also undertake follow up assessments. We feel the GP is well placed to undertake these interactions. They involve clinical aspects typically undertaken by a GP, such as assessment of co-morbidities, and reviewing for dementia and malnutrition; as well as providing study information, seeking informed consent and outlining the risks and benefits of the interventions.

 The interactions will be standardised across sites by ensuring co-investigators have a clear understanding of the protocol steps. Three preparatory (online) meetings have been held with all co-investigators to agree a standardised assessment approach (page 7). These have involved dry-runs/ role playing of participant interactions. Monthly meetings with all co-investigators will take place (page 7). These monthly meetings provide an opportunity to calibrate our approach in line with shared real assessment experiences. Additional ad-hoc meetings will enable real time communication on any challenges or questions that may arise and ensure the team remains aligned to a standardised approach.

 3) The Irish College of General Practitioners (ICGP) ethics committee advised that this time duration be applied. It is our understanding that the length of time for holding data may be set by the relevant authority overseeing the work. This approach is described by the UK Medical Research Council. The rationale is to ensure sufficient time is given to enable further research and publication subsequent to the trial, while adhering to GDPR regulations. We have amended the protocol to reflect this (page 8).

 Thank you for all your feedback and encouragement. It is hugely appreciated and will help us to progress our work.

# Peer review: Reviewer response for version 1

**DOI:** 10.21956/hrbopenres.14324.r28581

**Published:** 2021-01-11

**Authors:** Nicolas Martinez-Velilla

**Affiliations:** 1 Navarrabiomed, Complejo Hospitalario de Navarra (CHN), Navarra Health Research Institute (IdisNa), Public University of Navarra (UPNA), Navarra, Spain

• Is the rationale for, and objectives of, the study clearly described? Yes

 • Is the study design appropriate for the research question? Yes 

 • Are sufficient details of the methods provided to allow replication by others? Mostly (they have to describe which device they use for impendenciometry)

 This is an interesting article that presents a protocol for a randomized controlled trial using resistance exercises and dietary protein in order to reduce the level of frailty of the older adults in the community.

 As the authors write, although we are aware of the benefits of both interventions, it is often difficult to transfer and implement them in routine clinical practice.

 The article is well written and makes good use of current evidence, also highlighting that the authors start from a systematic review and meta-analysis that they have previously carried out. The methodological and statistical aspects are correct, with a good calculation of the sample size and a correct approach to statistical interpretation.

 I would like to make some / comments to the authors:

- A period of 3 months is sometimes short to show changes in situations of frailty, perhaps if they extend the follow-up period (although the intervention is not direct) it is worth it.
- The use of bioimpedance has certain limitations that I think should be reflected, and also it is necessary to describe what instruments will be used.
- I miss some aspects of CGA such as Barthel, Lawton, comorbidity or polypharmacy, and some geriatric syndrome (falls)…
- Perhaps it would be interesting to evaluate SPPB or gait speed, although I understand that they are time-consuming variables, but perhaps they are more sensitive to intervention.
- Regarding the exercise recommended by the authors, I think there is scientific evidence to advise multicomponent exercise and not just resistance, and instead of moderate activity HIIT exercises. An example could be the resources that you can find in http://vivifrail.com/ that allows the intervention to be highly individualized; on the other hand, the exercise proposed by the authors will be the same for everyone and should be more personalized or individualized.
- I really liked the role of patients and institutions (PPI) in the development of the protocol, it is something that geriatricians should learn and implement. Perhaps the gender perspective must also be addressed in future studies.
- Another possible additional intervention that has been shown to be useful could be the adjustment of polypharmacy and in parallel, a possible outcome could be what happens with the number /type of drugs.
- Is the intervention able of modifying cognitive parameters? In this hypothesis, we would have to consider initial cognitive evaluation and trajectory.
- If you are going to be advised and increment in protein intake, perhaps kidney function could be evaluated.
- When mixing exercise and nutrition, we will wonder which component has more weight in the results, or if we only owe it to one of them, or if both are enhanced. In this case, each intervention could be stratified to see what the effect of each one is, or randomized by blocks.

My congratulations on the project, I hope that everything goes well and that my comments can be useful.

Is the study design appropriate for the research question?

Yes

Is the rationale for, and objectives of, the study clearly described?

Yes

Are sufficient details of the methods provided to allow replication by others?

Yes

Are the datasets clearly presented in a useable and accessible format?

Not applicable

Reviewer Expertise:

geriatrics, frailty, polypharmacy, iatrogenia, nosocomial disability

I confirm that I have read this submission and believe that I have an appropriate level of expertise to confirm that it is of an acceptable scientific standard, however I have significant reservations, as outlined above.

# Response

**Published:** 2021-01-28

**Authors:** John Travers

**Affiliations:** University College Dublin, Ireland

Dear Dr Martinez-Velilla,

 I am so grateful for your review and the constructive feedback you have offered that will strengthen this paper. I will work with our team to address each of these points and I look forward to submitting a revised version soon. 

 Best wishes,

 John

# Response

**Published:** 2021-03-10

**Authors:** John Travers

**Affiliations:** University College Dublin, Ireland

Thank you for this very helpful feedback and commentary. It helps us strengthen our protocol. I have addressed each of the points below, made amendments to the protocol and look forward to any other feedback that may arise.

 1) We agree a longer follow up period would be worthwhile. We would like to consider an additional follow up at 12 months if feasible, as well as our 3-month follow up described. We have included a 12-month follow up in the updated protocol and outlined that it is subject to further ethics approval (page 8).

 We still look forward to analysing what changes might occur over a 3-month period. 15 of the 46 studies we reviewed in our systematic review of primary care frailty interventions had follow up periods of circa 3 months and many showed statistically significant outcomes. We also found in our own feasibility study, which involved 94 participants, that improvements to general health were reported within two months. If positive outcomes can be demonstrated for a short time period, they will underline the benefit of investing participant and medical professional time and effort in promoting such interventions to achieve early and meaningful results.

 2) We have added details about the type of device and limitations of bioimpedance to our protocol (pages 3 and 8). This is very helpful feedback which will help a future write up and publication offering. Below is a longer reflection on bioimpedance and limitations:

 Bioimpedance analysis (BIA) is a safe, non-invasive and rapid way to assess body composition. Studies show that BIA is a reasonably accurate method for estimating body composition ^1,2^. Accuracy is broadly in line with other gold standard measurements, with one study showing correlations with DXA of 0.90 and above for foot-to-hand single and multiple frequency BIA ^2^.

 However, limitations include a reduction in absolute accuracy introduced by variable factors such as body weight, hydration and recent exercise ^1,3^. BIA may be less accurate in obese people ^4^. High extracellular water and total body water may overestimate fat-free mass (muscle and bone) and underestimate fat mass in individuals with body mass indices greater than 34 kg/m ^2^. Dehydration may cause fat-free mass to be underestimated ^1^. High intensity exercise may also affect the accuracy of BIA readings ^1^. Such variability in factors may contribute to reduced repeatability and reproducibility of results ^5^.

 Given the limitations described above, Buchholz has proposed that BIA may be acceptable for monitoring changes in body composition within individuals over time, while less accurate for single measurements in individual patients ^6^.

 A final limitation to consider is relative cost. Though less expensive than alternatives such as DXA scanners, medical grade BIA devices cost several thousand euro and may not be cost effective in small primary care practices.

1. Dehghan M, Merchant AT. Is bioelectrical impedance accurate for use in large epidemiological studies? *Nutr J*. 2008;7:26. doi:10.1186/1475-2891-7-26
2. Demura S, Sato S. Comparisons of accuracy of estimating percent body fat by four bioelectrical impedance devices with different frequency and induction system of electrical current. *J Sports Med Phys Fitness*. 2015;55(1-2):68-75.
3. Mulasi U, Kuchnia AJ, Cole AJ, Earthman CP. Bioimpedance at the bedside: current applications, limitations, and opportunities. Nutrition in Clinical Practice, vol. 30, no. 2, pp. 180–193, 2015
4. Coppini LZ, Waitzberg DL, Campos ACL. Limitations and validation of bioelectrical impedance analysis in morbidly obese patients, Current Opinion in Clinical Nutrition and Metabolic Care: May 2005 - Volume 8 - Issue 3 - p 329-332
5. Naranjo-Hernández D, Reina-Tosina J, Min M. Fundamentals, Recent Advances, and Future Challenges in Bioimpedance Devices for Healthcare Applications. Journal of Sensors, vol. 2019, Article ID 9210258, 42 pages, 2019
6. Buchholz AC, Bartok C, Schoeller DA. The validity of bioelectrical impedance models in clinical populations. Nutrition in Clinical Practice. 19 (5): 433–46

We are using the Tanita RD 545 Segmental Body Composition Analyser. It applies dual frequency technology, which is the mainstay of medical grade products and achieves high level accuracy. Measurements that can be recorded include: weight; body mass index; total muscle mass; muscle mass of the arms and legs; muscle mass of the trunk; body fat content; body water content; base metabolic rate; bone weight; and ‘biological age’.

 We did also consider using DXA-scans to measure bone density and also apply a novel algorithm to estimating muscle mass. However, we decided that the additional (albeit small) radiation dose to participants, the cost and the logistical challenges (especially during a pandemic) rendered this option less appropriate.

 3) We tried to get the balance right between recording enough information to establish meaningful study metrics and the practical constraints of a 15-minute primary care consultation for baseline and follow up visits. We do record diagnosed co-morbidities, including recurrent falls, that have been coded in the participant’s patient file. Polypharmacy is picked up in twice annual ‘chronic disease management (CDM)’ consultations.

 4) We began to measure gait speed at the start of our feasibility study. However, we noted that a free space of even 4m long was not readily available in small primary care (PC) practices. It was both a time-consuming variable to measure, as you mention, but also not practical in the confined spaces available, with corridors shared by other patients entering and exiting consultations or other facilities. We do record reported changes to walking/ slowness in the SHARE-FI model.

 5) Thank you, we agree that multicomponent exercises and not just resistance can be beneficial to participants, as shown in evidence. Our exercise regime includes exercises that aim to improve flexibility and balance as well as strength. The exercise guide also includes walking for 20 minutes 3-4 times a week. We also include a component where participants increase the number of repetitions with time to increase exercise intensity.

 The Vivifrail site is very helpful in demonstrating how to personalise exercise regimes for different ability levels. It points to how we can build on our exercises with more individualised regimes at follow up. The Vivifrail exercises are described as supporting strength, flexibility, balance and cardiovascular health. Many are similar to the exercises we are proposing. Our regime of 10 exercises provides even more exercise options than the 6 to 8 exercises in the Vivifrail passports.

 6) Thank you, we wished to ensure that potential participants were involved in co-designing the trial to ensure their priorities were at its core. We will continue to incorporate gender perspectives in our future work.

 7) All primary care practices in Ireland now have a ‘chronic disease management (CDM)’ programme for older people. A key part of CDM is to review medications with every patient and address polypharmacy where appropriate. This dovetails well with our frailty assessment and intervention as the CDM consultation (twice yearly) is an opportunity to introduce this intervention. Polypharmacy review can be done in parallel. Changes in the number and types of drugs might be more suited to our 12-month follow up than the initial 3-month one.

 8) This is an excellent point. It would be very interesting to assess cognitive parameters. It is beyond the scope of this study but something we could address in future work.

 9) Following review of the literature on recommended levels of protein in chronic kidney disease (CKD), we note recent guidance of daily protein intake of 0.6-0.8g /kg body weight for those with CKD stage 3-4 (unless on dialysis). We have added CKD stage 3 or 4 to our exclusion criteria (page 7). Thank you for this helpful feedback.

Dietary protein intake and chronic kidney disease - PubMed (nih.gov)

 10) Our systematic review and meta-analysis give some insight into this, with exercise regimes shown to be more effective in delaying or reversing frailty.

 Randomising by blocks would increase the required sample size beyond what we can achieve in the time frame for this current study.

 We can seek input from our independent statistician on how best to analyse the data to derive additional insights on differences of adherence to the exercise and dietary components in relation to outcomes.

 Thank you for your constructive and helpful feedback.
